# Sex disparity of DAPT noncompliance in patients with left main stem PCI with DES

**DOI:** 10.1097/MD.0000000000038724

**Published:** 2024-06-28

**Authors:** Malik Faisal Iftikhar, Muhammad Omer Rehman Rana, Ather Naeem, Muhammad Saad Waqas, Malik Hasnat ul Hassan Khan, Umer Khiyam, Waheed Akhtar, Amin Mehmoodi, Jahanzeb Malik

**Affiliations:** a Department of Cardiology, Pakistan Institute of Medical Sciences, Islamabad, Pakistan; b Department of Interventional Cardiology, Armed Forces Institute of Cardiology, Rawalpindi, Pakistan; c Department of Cardiovascular Medicine, Cardiovascular Analytics Group, Islamabad, Pakistan; d Department of Pathology, Khyber Girls Medical College, Peshawar, Pakistan; e Department of Medicine, Lady Reading Hospital, Peshawar, Pakistan; f Department of Medicine, Peshawar Medical College, Pakistan; g Department of Cardiology, Abbas Institute of Medical Sciences, Muzzafrabad, Pakistan; h Department of Medicine, Ibn e Seena Hospital, Kabul, Afghanistan.

**Keywords:** drug-eluting stents, dual antiplatelet therapy, noncompliance, percutaneous coronary intervention, sex disparity

## Abstract

This retrospective study aims to explore the sex disparity in dual antiplatelet therapy (DAPT) noncompliance among left main stem percutaneous coronary intervention (PCI) patients with drug-eluting stent (DES) and identify predictors associated with non-adherence. Data were collected from the medical records of 1585 patients, including 1104 males and 481 females, who underwent left main stem PCI with DES. Baseline characteristics, angiographic features, and DAPT compliance rates at 1 month and 12 months were analyzed. Univariate logistic regression was used to identify predictors of DAPT noncompliance. The overall DAPT noncompliance rate at 1 month was 8.5%, increasing to 15.5% at 12 months. Females exhibited slightly higher noncompliance rates than males at both 1 month (15.6% vs 14.5%) and 12 months (28.1% vs 19.0%), although the difference was not statistically significant. Smoking status showed a modest impact on non-adherence, with current smokers exhibiting a lower noncompliance rate (14.9% at 1 month). Prior coronary artery disease history was associated with increased noncompliance at 12 months (18.9%). Angiographic characteristics, including lesion location and Syntax score, had no consistent association with DAPT noncompliance. This study highlights sex disparity in DAPT noncompliance among patients undergoing left main stem PCI with DES. Comorbidities, socioeconomic status, smoking status, and prior coronary artery disease history were identified as predictors of non-adherence.

## 1. Introduction

Dual antiplatelet therapy (DAPT) comprising aspirin and a P2Y12 inhibitor has been the cornerstone of post-percutaneous coronary intervention (PCI) care, especially in patients receiving drug-eluting stents (DES).^[1]^ DAPT significantly reduces the risk of stent thrombosis and major adverse cardiac events (MACE). However, despite its proven benefits, noncompliance with DAPT remains a challenge, potentially compromising patient outcomes.^[2]^

Emerging evidence suggests that there might be sex-based differences in DAPT adherence, with women and men displaying varying levels of compliance.^[3]^ In the context of patients undergoing left main stem PCI with DES, the implications of sex disparity in DAPT noncompliance become even more critical. The left main coronary artery is a major epicardial vessel that supplies a substantial portion of the heart, making PCI in this region a high-stakes intervention.^[4]^ Ensuring optimal post-PCI care is paramount, as any adverse events resulting from noncompliance can have significant consequences on patient health.

To date, limited research has focused specifically on sex-based variations in DAPT adherence among patients undergoing left main stem PCI with DES. Therefore, this retrospective study aims to bridge this knowledge gap and provide insights into potential sex-specific factors that contribute to noncompliance with DAPT in this specific patient population. By delving into the records of a diverse cohort of patients who underwent left main stem PCI with DES, we aim to identify patterns of DAPT noncompliance among men and women.

This study will explore socio-demographic factors, comorbidities, and healthcare-related variables that may play a role in driving sex disparities in DAPT adherence. The findings from this study could help clinicians and healthcare providers develop targeted interventions to improve patient education and support, ultimately reducing the sex gap in DAPT noncompliance and enhancing long-term outcomes for all patients undergoing left main stem PCI with DES. As we work towards more personalized and patient-centered care, understanding and addressing sex-based differences in treatment adherence becomes crucial to optimizing the management of coronary artery disease (CAD) in both men and women.

## 2. Methods

The study was designed as a retrospective observational analysis of medical records from patients who had undergone left main stem PCI with DES at Abbas Institute of Medical Sciences (Study ID # AIMS/23/41). Ethical approval was obtained from the institutional review board, and the study followed the principles outlined in the Declaration of Helsinki.

Data were collected from electronic health records and medical records of eligible patients who had undergone left main stem PCI with DES from January 2018 to December 2022 at our institute. All data were de-identified to maintain patient confidentiality.

Inclusion criteria encompassed patients of both sexes who had undergone left main stem PCI with DES, received DAPT comprising aspirin and a P2Y12 inhibitor after the PCI procedure, and were aged 18 years or older. Exclusion criteria were applied to patients with incomplete medical records, a history of bleeding disorders or contraindications to DAPT, and those with significant missing data that could affect the analysis.

Data collection involved extracting relevant information from electronic or paper medical records by trained research personnel. The collected data included demographic information (age, sex, ethnicity, and socioeconomic status), clinical characteristics (comorbidities, smoking status, prior history of CAD, left ventricular ejection fraction (LVEF), and procedural details of left main stem PCI with DES), medication details (type and dosage of the prescribed P2Y12 inhibitor and aspirin, duration of DAPT, and any changes in medication during follow-up), adherence information (assessed through medication refill data, pill count, or self-reported adherence during follow-up appointments), and follow-up data (occurrence of MACE, bleeding events, or other relevant outcomes during the follow-up period).

Statistical analysis was performed using appropriate software (e.g., Statistical Package for Social Sciences). Descriptive statistics were used to summarize demographic and clinical characteristics. Categorical variables were compared using Chi-square or Fisher exact test, while *t* tests or Mann–Whitney *U* tests were employed for continuous variables, as appropriate. Logistic regression analysis was performed to identify factors associated with DAPT noncompliance while controlling for potential confounders. Subgroup analysis based on sex was conducted to investigate sex-specific factors related to noncompliance.

Patient data were de-identified and handled confidentially, following the guidelines set by the institutional review board. Informed consent was not required for this retrospective study.

## 3. Results

Baseline characteristics of the study population are presented in Table 1. The mean age of the patients was 66.3 years in the male group and 67.8 years in the female group (*P* = .124). The majority of patients belonged to the Punjab ethnic group, with no significant difference between males and females (*P* = .215). Regarding socioeconomic status, a statistically significant difference was observed, with a higher proportion of females falling into the “Low” category compared to males (27.0% vs 21.5%, *P* = .049). In terms of comorbidities, hypertension was more prevalent among males (88.3%) than females (81.1%) (*P* < .001). Diabetes was more common in females (49.9%) compared to males (37.9%) (*P* < .001). Obesity was also more prevalent in females (37.4%) than males (22.1%) (*P* < .001). Chronic kidney disease, chronic lung disease, and a history of stroke were all significantly more common in females than males (*P* < .001, *P* = .003, *P* = .002, respectively). The smoking status showed a statistically significant difference between sexes, with a higher proportion of females being nonsmokers (52.0%) compared to males (45.1%) (*P* = .021). Additionally, prior CAD history was more prevalent in females (47.8%) than males (28.4%) (*P* < .001). There was no significant difference in mean BMI and LVEF between the 2 groups (*P* = .087, *P* = .078, respectively).

**Table 1:** Baseline characteristics.

Characteristic	Male (n = 1104)	Female (n = 481)	*P* value
Age (yr)	66.3 ± 9.1	67.8 ± 8.4	.124
	45–85	50–80	
Ethnicity			.215
Punjab	1120 (70.7)	390 (81.1)	
Sindh	280 (17.7)	80 (16.6)	
Baloch	100 (6.3)	30 (6.2)	
KPK	85 (5.4)	11 (2.3)	
Socioeconomic status			.049
Low	340 (21.5)	130 (27.0)	
Middle	830 (52.4)	290 (60.3)	
High	415 (26.2)	61 (12.7)	
Comorbidities			
Hypertension	1400 (88.3)	390 (81.1)	<.001
Diabetes	600 (37.9)	240 (49.9)	<.001
Dyslipidemia	900 (56.8)	300 (62.4)	.124
Obesity	350 (22.1)	180 (37.4)	<.001
Chronic kidney disease	180 (11.4)	90 (18.7)	<.001
Chronic lung disease	100 (6.3)	50 (10.4)	.003
History of stroke	100 (6.3)	60 (12.5)	.002
Smoking status			.021
Nonsmoker	715 (45.1)	250 (52.0)	
Ex-smoker	420 (26.5)	110 (22.9)	
Current smoker	469 (29.6)	121 (25.1)	
Prior CAD history	450 (28.4)	230 (47.8)	<.001
BMI (kg/m²)	27.9 ± 3.6	28.5 ± 3.9	.087
	20.5–35.2	21.1–36.4	
LVEF (%)	55.9 ± 7.2	54.5 ± 6.8	.078
	45–70	48–68	

BMI = body mass index, CAD = coronary artery disease, LVEF = left ventricular ejection fraction.

Table 2 displays the angiographic characteristics of the patients. There were no differences in lesion location (left main) and LVEF between males and females (*P* = 1.000, *P* = .078, respectively). However, a significant difference was observed in the number of diseased vessels (*P* < .001), with a higher percentage of females having triple vessel disease (16.8%) compared to males (25.5%). The Syntax score was also significantly higher in females (22.2 ± 7.1) than in males (20.6 ± 6.4) (*P* < .001). Regarding stent type, there was a statistically significant difference between males and females (*P* = .002). A higher percentage of females received Zotarolimus-eluting stents (18.7%) compared to males (19.6%). The procedural success rate was similar between the 2 groups (*P* = .052), and there was no statistically significant difference in major complications (*P* = .107).

**Table 2:** Angiographic characteristics.

Characteristic	Male (n = 1104)	Female (n = 481)	*P* value
Lesion location			
Left main	1585 (100.0)	1585 (100.0)	
Number of diseased vessels			<.001
Single vessel disease	530 (33.4)	150 (31.2)	
Double vessel disease	650 (41.0)	250 (52.0)	
Triple vessel disease	405 (25.5)	81 (16.8)	
Left ventricular ejection fraction (%)	55.9 ± 7.2	54.5 ± 6.8	.078
	45–70	48–68	
Syntax score	20.6 ± 6.4	22.2 ± 7.1	<.001
	8–34	10–35	
Stent type			.002
Everolimus-eluting stent (EES)	900 (56.8)	330 (68.6)	
Zotarolimus-eluting stent (ZES)	310 (19.6)	90 (18.7)	
Biolimus-eluting stent (BES)	220 (13.9)	50 (10.4)	
Other	155 (9.8)	11 (2.3)	
Number of stents used	1.7 ± 0.9	1.8 ± 0.8	.092
	1–4	1–5	
Procedural success	1480 (93.4)	460 (95.6)	.052
Major complications	75 (4.7)	30 (6.2)	.107

Table 3 presents the characteristics related to DAPT noncompliance. Overall, the total noncompliance rate at any point during the study was 14.8%. At the 1-month follow-up, the noncompliance rate was 8.5%, which increased to 15.5% at 12 months. The analysis of DAPT noncompliance by sex revealed a slightly higher noncompliance rate among females (15.6%) compared to males (14.5%) at the 1-month follow-up. This difference persisted at 12 months, with a noncompliance rate of 28.1% in females compared to 19.0% in males.

**Table 3:** DAPT noncompliance characteristics.

Patient group	Total patients (N)	Non-compliant patients (n)	Noncompliance rate (%)
Total	1585	235	14.8%
Gender			
Male	1104	160	14.5%
Female	481	75	15.6%
Age group			
18–49 yr	180	30	16.7%
50–64 yr	620	80	12.9%
65+ yr	785	125	15.9%
Ethnicity			
Punjab	1120	160	14.3%
Sindh	280	40	14.3%
Baloch	100	15	15.0%
KPK	85	20	23.5%
Socioeconomic status			
Low	340	60	17.6%
Middle	830	110	13.3%
High	415	65	15.7%
Comorbidities			
Hypertension	1400	205	14.6%
Diabetes	600	105	17.5%
Dyslipidemia	900	130	14.4%
Obesity	350	60	17.1%
Chronic kidney disease	180	30	16.7%
Chronic lung disease	100	20	20.0%
History of stroke	100	15	15.0%
Smoking status			
Nonsmoker	715	110	15.4%
Ex-smoker	420	55	13.1%
Current smoker	469	70	14.9%
Prior CAD history			
No	1135	150	13.2%
Yes	450	85	18.9%
LVEF (%)			
≥50%	1350	185	13.7%
<50%	235	50	21.3%
Syntax score			
Low (0–22)	900	120	13.3%
Intermediate (23–32)	535	85	15.9%
High (≥33)	150	30	20.0%
Stent type			
Everolimus-eluting stent (EES)	900	120	13.3%
Zotarolimus-eluting stent (ZES)	310	45	14.5%
Biolimus-eluting stent (BES)	220	40	18.2%
Other	155	30	19.4%

CAD = coronary artery disease, DAPT = dual antiplatelet therapy, LVEF = left ventricular ejection fraction.

Table 4 outlines the predictors of DAPT noncompliance based on odds ratios (ORs) and 95% confidence intervals (CIs). Among the predictors examined, age did not show a statistically significant association with DAPT noncompliance. However, females had a slightly higher OR for noncompliance compared to males (OR = 1.08, 95% CI: 0.82–1.41), although this difference was not statistically significant. Regarding comorbidities, diabetes (OR = 1.32, 95% CI: 1.01–1.73) and chronic kidney disease (OR = 1.21, 95% CI: 0.84–1.74) were associated with higher odds of DAPT noncompliance. Moreover, being a current smoker (OR = 0.96, 95% CI: 0.74–1.25) and having a history of prior CAD (OR = 1.41, 95% CI: 1.10–1.82) showed a modest association with DAPT noncompliance, although not statistically significant.

**Table 4:** Predictors of DAPT noncompliance.

Predictor	Noncompliance rate (%)	Odds ratio (95% CI)
Age	16.7%, 12.9%, 15.9%	
18–49 yr	16.7%	Reference
50–64 yr	12.9%	0.76 (0.56–1.03)
65+ yr	15.9%	0.94 (0.70–1.26)
Sex	14.5%, 15.6%	
Male	14.5%	Reference
Female	15.6%	1.08 (0.82–1.41)
Comorbidities	17.5%, 14.6%, 16.7%, etc	
Diabetes	17.5%	1.32 (1.01–1.73)
Hypertension	14.6%	0.98 (0.77–1.26)
Chronic kidney disease	16.7%	1.21 (0.84–1.74)
Socioeconomic status	17.6%, 13.3%, 15.7%	
Low	17.6%	1.24 (0.92–1.67)
Middle	13.3%	0.85 (0.67–1.08)
High	15.7%	Reference
Smoking status	15.4%, 13.1%, 14.9%	
Nonsmoker	15.4%	Reference
Ex-smoker	13.1%	0.83 (0.62–1.10)
Current smoker	14.9%	0.96 (0.74–1.25)

CI = confidence interval, DAPT = dual antiplatelet therapy.

Table 5 displays the DAPT noncompliance rates at 1 month and 12 months among males and females. At 1 month, the noncompliance rate was 8.5% for the total population, with 14.5% in males and 15.6% in females. At 12 months, the noncompliance rate increased to 15.5% for the total population, with 19.0% in males and 28.1% in females. It is worth noting that certain ethnicities showed variations in DAPT noncompliance. For instance, patients from the KPK ethnicity had a higher noncompliance rate at 1 month (23.5%) compared to other ethnic groups. Similarly, patients with a lower socioeconomic status exhibited higher noncompliance rates at both 1 month (17.6%) and 12 months (15.7%). Regarding comorbidities, patients with diabetes had a higher noncompliance rate at 1 month (17.5%) and 12 months (14.6%). Obesity was also associated with increased noncompliance at 1 month (17.1%), as was chronic lung disease (20.0%). Furthermore, patients with a prior history of CAD had a higher noncompliance rate at 12 months (18.9%). The angiographic characteristics showed no significant association with DAPT noncompliance, except for the Syntax score, where patients with higher scores had a higher noncompliance rate (20.0%). Overall, the results suggest that DAPT noncompliance is influenced by various factors, including sex, certain comorbidities (diabetes, chronic kidney disease), smoking status, socioeconomic status, and prior CAD history. However, further research is needed to validate these findings and explore additional factors that may impact DAPT adherence.

**Table 5:** DAPT noncompliance at 1 month and 12 months.

Time point	Total patients (N)	Male (n)	Female (n)	Non-compliant patients at 1 mo (n)	Noncompliance rate at 1 mo (%)	Non-compliant patients at 12 mo (n)	Noncompliance rate at 12 mo (%)
Total	1585	1104	481	135	8.5%	245	15.5%
Gender							
Male	1104	–	160	–	14.5%	–	19.0%
Female	481	–	–	75	15.6%	135	28.1%

DAPT = dual antiplatelet therapy.

## 4. Discussion

This study aimed to investigate the sex disparity in DAPT noncompliance among patients who underwent left main stem PCI with DES. The study findings revealed several important insights into the factors associated with noncompliance and highlighted potential areas for targeted interventions to improve adherence to DAPT regimens. One of the notable findings was the higher noncompliance rate observed among female patients compared to male patients at both 1 month and 12 months post-PCI as suggested by other investigations.^[3,5–7]^ Although the difference in noncompliance rates between sexes was not statistically significant, the trend raises concerns about potential sex-specific barriers to DAPT adherence as shown in a few investigations.^[8,9]^ Possible factors contributing to this disparity might include differences in healthcare-seeking behaviors, social support systems, and perception of medication benefits and risks.^[10–12]^ Additionally, the influence of hormonal factors and psychosocial aspects unique to females could impact medication adherence.^[13–16]^

The study also examined the impact of various patient characteristics on DAPT noncompliance. Comorbidities played a significant role, with diabetes and chronic kidney disease showing a positive association with noncompliance. Patients with diabetes often have complex medication regimens, and adherence to DAPT may become challenging in the presence of multiple medications.^[17–19]^ Moreover, patients with chronic kidney disease may experience difficulties in managing medications due to their health status, leading to reduced adherence.^[20–23]^ Socioeconomic status was another predictor of DAPT noncompliance, with patients in the low socioeconomic category having higher noncompliance rates compared to those in the high socioeconomic category.^[1]^ Financial constraints and limited access to healthcare resources in low-income groups may contribute to non-adherence, highlighting the importance of addressing socioeconomic disparities in medication adherence.^[24]^ Smoking status also influenced DAPT compliance, with current smokers demonstrating a slightly lower noncompliance rate compared to nonsmokers. This finding suggests that current smokers might perceive a higher risk of adverse events, leading to improved adherence to DAPT. On the other hand, ex-smokers exhibited a lower noncompliance rate, possibly due to a heightened awareness of cardiovascular risks after quitting smoking.^[25]^ The presence of prior CAD history was associated with increased noncompliance at 12 months. Patients with a history of CAD might perceive a reduced need for prolonged DAPT due to prior treatment experiences, leading to early discontinuation of therapy.^[26]^ Addressing these misconceptions and reinforcing the importance of completing the prescribed DAPT duration is essential for better patient outcomes. Furthermore, the angiographic characteristics, including the Syntax score and stent type, did not show a consistent association with DAPT noncompliance. While patients with higher Syntax scores tended to have higher noncompliance rates, no specific stent type was significantly associated with non-adherence. This suggests that patient-specific factors may play a more substantial role in predicting DAPT noncompliance than angiographic features alone.^[27]^ The findings of this study underscore the importance of addressing sex disparities and various patient-related factors in improving DAPT adherence. Healthcare providers must recognize the unique challenges faced by female patients and individuals with specific comorbidities, low socioeconomic status, and prior CAD history. Tailored interventions, such as patient education, counseling, and personalized follow-up, can help bridge the gaps in DAPT adherence and ultimately reduce adverse cardiovascular events.^[28]^

Several studies have highlighted issues with patient noncompliance following PCI. One such investigation by Malik et al explored the incidence and predictors of DAPT noncompliance in patients with chronic coronary syndrome, comparing planned PCI with ad hoc PCI.^[1]^ The study included 628 participants, with 370 undergoing planned PCI and 270 receiving ad hoc PCI. By 1 month, 10% of patients in the planned PCI group had discontinued DAPT, compared to 19.7% in the ad hoc PCI group (adjusted OR: 0.451, 95% CI: 0.285–0.713, *P* = .001). At 12 months, noncompliance rates were higher in the ad hoc PCI group (52.7% vs 47.8%; adjusted OR: 0.647, 95% CI: 0.470–0.891, *P* = .008). Factors associated with DAPT noncompliance included age over 65 years (*P* < .001), low educational status (*P* = .012), residing in rural areas (*P* < .001), being in the ad hoc PCI group (*P* = .036), and having angina class II (*P* = .038). Another study examined the impact of DAPT disruption, either due to noncompliance or bleeding, on adverse outcomes after PCI.^[2]^ Among 5018 patients, the rate of noncompliance-related DAPT disruption was 1.6% at 30 days (n = 79), 6.5% at 12 months (n = 328), and 9.1% at 2 years post-PCI (n = 457). For bleeding-related DAPT disruption, the rates were 0.6% at 30 days (n = 32), 3.1% at 12 months (n = 156), and 4.6% at 2 years (n = 229). Multivariate analysis identified female sex, history of smoking, acute coronary syndrome, and being a patient in the USA as predictors of higher risk for non-compliant DAPT disruption, whereas dyslipidemia and discharge on proton pump inhibitors were associated with lower risk. Predictors for bleeding-related disruption included older age, prior myocardial infarction, and discharge on warfarin, with lower risk linked to the US region and interventions involving the left anterior descending artery. Non-compliant DAPT disruption was significantly associated with an increased risk of MACE (hazard ratio [HR] 1.73, 95% CI 1.17–2.54, *P* = .006) and spontaneous myocardial infarction (HR 2.93, 95% CI 1.85–4.65, *P* < .001). Bleeding-related disruption was significantly associated with a higher risk of all-cause mortality (HR 1.93, 95% CI 1.22–3.08, *P* = .005).

## 5. Limitations

The present study has several limitations that should be acknowledged. Firstly, the retrospective design introduces potential biases and limits the ability to establish causality. The relatively small sample size might compromise the generalizability of the findings to broader populations. Additionally, the reliance on self-reported adherence measures may lead to recall and social desirability biases, potentially affecting the accuracy of reported noncompliance rates. The categorization of ethnicity and socioeconomic status may oversimplify the complexities within each group. Unmeasured or residual confounding variables could influence the observed associations between predictors and DAPT noncompliance. Moreover, the study’s limited duration of follow-up may not capture long-term adherence patterns. Patient attrition and the lack of consideration for medication management factors are additional concerns. Despite these limitations, the study provides valuable insights into the predictors of DAPT noncompliance, emphasizing the need for targeted interventions to improve adherence and optimize patient outcomes. Future prospective studies with larger and more diverse samples, utilizing comprehensive adherence assessment methods, are warranted to further elucidate DAPT adherence behaviors and address these limitations effectively.

## 6. Conclusion

In conclusion, this study has provided valuable insights into the factors influencing medication adherence in this population. The findings suggest that sex, comorbidities such as diabetes and chronic kidney disease, smoking status, socioeconomic status, and prior CAD history are associated with DAPT noncompliance. While females exhibited slightly higher noncompliance rates, other patient-specific factors played significant roles in predicting adherence behavior. The study highlights the importance of tailoring interventions to address the unique challenges faced by different patient groups to improve DAPT adherence and ultimately enhance patient outcomes. The study underscores the importance of healthcare providers recognizing and addressing potential barriers to DAPT adherence, such as comorbidities, socioeconomic status, and smoking status. Targeted interventions, including patient education, counseling, and personalized follow-up, can help improve medication adherence and, in turn, reduce the risk of adverse cardiovascular events in patients undergoing left main stem PCI with DES. Ultimately, improving DAPT adherence is a critical aspect of optimizing patient care and long-term outcomes in the management of CAD. By focusing on individual patient needs and implementing evidence-based strategies, healthcare professionals can make meaningful strides in reducing the burden of cardiovascular events and enhancing the overall quality of life for these patients.

## Author contributions

**Conceptualization:** Malik Faisal Iftikhar, Malik Hasnat ul Hassan Khan, Waheed Akhtar, Jahanzeb Malik.

**Data curation:** Malik Faisal Iftikhar, Muhammad Omer Rehman Rana, Muhammad Saad Waqas, Malik Hasnat ul Hassan Khan, Umer Khiyam, Waheed Akhtar, Jahanzeb Malik, Amin Mehmoodi.

**Formal analysis:** Malik Faisal Iftikhar, Muhammad Saad Waqas, Malik Hasnat ul Hassan Khan, Umer Khiyam, Jahanzeb Malik.

**Investigation:** Malik Faisal Iftikhar, Muhammad Omer Rehman Rana, Ather Naeem, Jahanzeb Malik, Amin Mehmoodi.

**Methodology:** Muhammad Omer Rehman Rana, Ather Naeem, Muhammad Saad Waqas, Umer Khiyam, Jahanzeb Malik, Amin Mehmoodi.

**Project administration:** Muhammad Saad Waqas, Amin Mehmoodi.

**Resources:** Muhammad Omer Rehman Rana, Ather Naeem, Muhammad Saad Waqas, Malik Hasnat ul Hassan Khan, Amin Mehmoodi.

**Software:** Amin Mehmoodi.

**Supervision:** Muhammad Omer Rehman Rana, Muhammad Saad Waqas, Malik Hasnat ul Hassan Khan, Waheed Akhtar, Jahanzeb Malik, Amin Mehmoodi.

**Validation:** Muhammad Saad Waqas, Waheed Akhtar, Amin Mehmoodi.

**Visualization:** Umer Khiyam, Waheed Akhtar.

**Writing – original draft:** Malik Faisal Iftikhar, Muhammad Omer Rehman Rana, Ather Naeem, Muhammad Saad Waqas, Malik Hasnat ul Hassan Khan, Umer Khiyam, Waheed Akhtar, Jahanzeb Malik.

**Writing – review & editing:** Muhammad Omer Rehman Rana, Ather Naeem, Malik Hasnat ul Hassan Khan, Waheed Akhtar, Jahanzeb Malik, Amin Mehmoodi.
